# Deficient Liver Biosynthesis of Docosahexaenoic Acid Correlates with Cognitive Impairment in Alzheimer's Disease

**DOI:** 10.1371/journal.pone.0012538

**Published:** 2010-09-08

**Authors:** Giuseppe Astarita, Kwang-Mook Jung, Nicole C. Berchtold, Vinh Q. Nguyen, Daniel L. Gillen, Elizabeth Head, Carl W. Cotman, Daniele Piomelli

**Affiliations:** 1 Department of Pharmacology, University of California Irvine, Irvine, California, United States of America; 2 Department of Experimental Medicine and Biochemical Sciences, University of Rome Tor Vergata, Rome, Italy; 3 Institute for Brain Aging and Dementia, University of California Irvine, Irvine, California, United States of America; 4 Department of Statistics, University of California Irvine, Irvine, California, United States of America; 5 Sanders-Brown Center on Aging, University of Kentucky, Lexington, Kentucky, United States of America; 6 Department of Biological Chemistry, University of California Irvine, Irvine, California, United States of America; 7 Unit of Drug Discovery and Development, Italian Institute of Technology, Genoa, Italy; Sapienza University of Rome, Italy

## Abstract

Reduced brain levels of docosahexaenoic acid (C22:6n-3), a neurotrophic and neuroprotective fatty acid, may contribute to cognitive decline in Alzheimer's disease. Here, we investigated whether the liver enzyme system that provides docosahexaenoic acid to the brain is dysfunctional in this disease. Docosahexaenoic acid levels were reduced in temporal cortex, mid-frontal cortex and cerebellum of subjects with Alzheimer's disease, compared to control subjects (P = 0.007). Mini Mental State Examination (MMSE) scores positively correlated with docosahexaenoic/α-linolenic ratios in temporal cortex (P = 0.005) and mid-frontal cortex (P = 0.018), but not cerebellum. Similarly, liver docosahexaenoic acid content was lower in Alzheimer's disease patients than control subjects (P = 0.011). Liver docosahexaenoic/α-linolenic ratios correlated positively with MMSE scores (r = 0.78; P<0.0001), and negatively with global deterioration scale grades (P = 0.013). Docosahexaenoic acid precursors, including tetracosahexaenoic acid (C24:6n-3), were elevated in liver of Alzheimer's disease patients (P = 0.041), whereas expression of peroxisomal d-bifunctional protein, which catalyzes the conversion of tetracosahexaenoic acid into docosahexaenoic acid, was reduced (P = 0.048). Other genes involved in docosahexaenoic acid metabolism were not affected. The results indicate that a deficit in d-bifunctional protein activity impairs docosahexaenoic acid biosynthesis in liver of Alzheimer's disease patients, lessening the flux of this neuroprotective fatty acid to the brain.

## Introduction

Alzheimer's disease is a neurodegenerative disorder characterized clinically by progressive cognitive impairment [1]. Age is the most important factor that predisposes persons to the non-familial form of the disease, which in 2010 affected over 35 million elderly adults worldwide [2]. How aging interacts with other risk factors for Alzheimer's disease [3] is still unknown. It appears, however, that certain age-related pathologies that are closely associated with systemic dysfunctions in lipid metabolism – including obesity and diabetes – might be involved [1].

The polyunsaturated lipid, docosahexaenoic acid (C22:6n-3), is an essential component of neuronal membranes [4], [5] and a precursor for potent neuroprotective mediators [6]–[8]. Mammals obtain docosahexaenoic acid directly from dietary sources, especially fish, but can also produce it in liver from n-3 fatty acid precursors present in plants [9]–[11]. When the diet does not provide an adequate supply of these foods, as is often the case in contemporary populations [12], the liver's capacity to generate docosahexaenoic acid may become critical to keep normal the brain levels of this fatty acid [9], [11], [13], [14].

**Figure 1:**
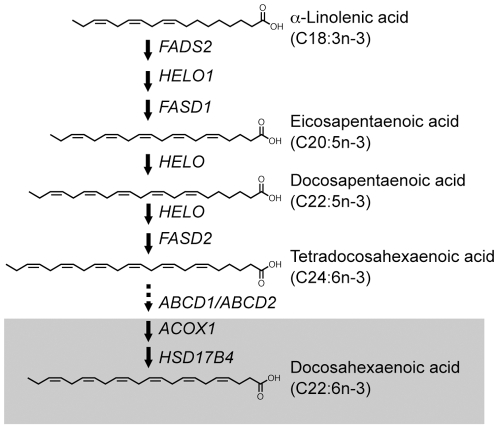
Overview of docosahexaenoic acid biosynthesis in liver. Diet-derived α-linolenic acid (C18:3n-3) is transformed into tetracosahexaenoic acid (C24:6n-3) by the sequential action of Δ^6^ and Δ^5^ desaturases (encoded by the *FADS2* and *FADS1* genes, respectively) and elongases (such as that encoded by the *HELO1* gene) present in the endoplasmatic reticulum. Tetracosahexaenoic acid is transported into peroxisomes (shaded area), presumably by proteins encoded by the *ABCD1* or *ABCD2* genes, and then converted into docosahexaenoic acid (C22:6n-3) by sequential action of acyl coenzyme-A oxidase (encoded by the *ACOX1* gene), d-bifunctional protein (encoded by the *HSD17B4* gene), and various peroxisomal thiolases (not shown). The figure shows chemical structures of fatty acids quantified in our analyses.

Figure 1 shows an overview of liver docosahexaenoic acid biosynthesis. Elongase and desaturase enzymes localized in the endoplasmic reticulum of the hepatocyte progressively add carbon units and double bonds to shorter-chain n-3 fatty acids, producing the very-long-chain tetracosahexaenoic acid (C24:6n-3). This is transported into peroxisomes and then converted to docosahexaenoic acid by the sequential action of acyl-coenzyme A oxidases, d-bifunctional protein and peroxisomal thiolases [15]–[18]. Liver-derived docosahexaenoic acid reaches the brain through the circulation, probably bound to proteins that are also synthesized by hepatocytes [9].

Evidence indicates that docosahexaenoic acid serves important neurotrophic functions during early mammalian development [19], particularly as precursors of phospholipids during synaptogenesis, but is still unclear whether it plays protective roles in adulthood and old age. Several, albeit not all, epidemiological and clinical studies suggest that higher intake of docosahexaenoic acid decreases the risk of cognitive decline and dementia in elderly adults [4]. Animal experiments support this conclusion [20]–[22] and further indicate that the fatty acid might exert these effects by promoting neuronal survival [7], [23]. The related question of whether alterations in brain docosahexaenoic acid levels might accompany cognitive decline has been addressed using post mortem brain tissue from Alzheimer's disease patients and age-matched control subjects [6], [24]–[30]. Despite some disparities, these investigations generally support the hypothesis that Alzheimer's disease may be associated with deficits in brain docosahexaenoic acid [31]. In the present study, we reexamined this possibility and searched for supporting correlative evidence that a failing in brain docosahexaenoic acid integrity might result from defective n-3 fatty acid metabolism in liver, as previously suggested by Scott and Bazan [9].

## Results

### Docosahexaenoic acid levels in brain

Brain levels of non-esterified (‘free’) docosahexaenoic acid were measured in extracts of temporal cortex, mid-frontal cortex and cerebellum from a total of 17 control subjects and 37 Alzheimer's disease patients. Table 1 shows estimated mean differences between the two groups after linear regression adjustment for age, gender and post mortem interval. There were statistically detectable differences (P<0.05) in docosahexaenoic acid content in all regions examined. The pooled adjusted difference between control subjects and Alzheimer's disease patients (95% confidence intervals) was −28.87 nanomoles per gram of tissue (−49.81, −7.94; P = 0.007).

**Table 1:** Levels of free docosahexaenoic acid (nmol/g) and docosahexaenoate-containing phosphatidylethanolamine (nmol/g) in various brain regions of control subjects and subjects with Alzheimer's disease.

	Control subjects	Subjects with Alzheimer's disease	Adjusted Difference	P-value*
	Mean ± SD ; N	Mean ± SD ; N	(95% CI)	
DHA				
Temporal cortex	123.61±24.07 ; 17	102.29±40.66 ; 36	−23.87 ( −45.18, −2.57 )	0.029
Frontal cortex	119.99±50.8 ; 17	97.45±26.19 ; 37	−22.11 ( −42.37, −1.85 )	0.033
Cerebellum	218.18±101.66 ; 16	174.86±45.22 ; 35	−42.58 ( −83.31, −1.84 )	0.041
Pooled	152.64±79.12 ; 17	124.35±51.72 ; 37	−28.87 ( −49.81, −7.94 )	0.007
Phosphatidylethanolamine**				
Temporal cortex	11.52±3.58 ; 17	8.26±4.06 ; 36	−3.22 ( −5.56, −0.88 )	0.008
Frontal cortex	29.7±5.94 ; 17	23.83±5.84 ; 37	−5.82 ( −9.32, −2.32 )	0.002
Cerebellum	13.71±4.16 ; 16	10.37±4.75 ; 35	−3.44 ( −6.28, −0.61 )	0.018
Pooled	18.4±9.43 ; 17	14.32±8.6 ; 38	−4.14 ( −7.2, −1.09 )	0.008

Abbreviations: CI, confidence interval.

*P-values for differences between means were computed by linear regression analysis for each fatty acid in selected brain regions and Generalized Estimating Equations for the pooled analysis in the entire brain after adjustment for age, gender, and post mortem interval.

**Phosphatidylethanolamine was 1-stearoyl, 2-docosahexaenoyl-*sn*-glycero-phosphoethanolamine.

Since docosahexaenoic acid is stored in membrane phospholipids, Table 1 also shows levels of docosahexaenoic acid-containing 1-stearoyl, 2-docosahexaenoyl-*sn*-glycero-3-phosphoethanolamine in the three brain regions. There were significant differences between control subjects and Alzheimer's disease patients in all regions. The pooled adjusted difference between the two groups was −4.14 (−7.20, −1.09; P = 0.008).

Levels of four n-3 fatty acids that serve as metabolic precursors for docosahexaenoic acid – α-linolenic acid (C18:3n-3), eicosapentaenoic acid (C20:5n-3), docosapentaenoic acid (C22:5n-3) and tetracosahexaenoic acid (C24:6n-3) – were quantified in the same extracts. There were marginally statistically detectable differences between control subjects and Alzheimer's disease patients only for mid-frontal cortex α-linolenic acid (P = 0.045) (Table 2). However, a significant difference between the two groups was detected when α-linolenic acid content was pooled across brain regions. No differences were observed for eicosapentaenoic acid and docosapentaenoic acid. Tetracosahexaenoic acid levels were below the detection limit of our assay (0.5 picomoles per sample).

**Table 2:** Levels of free n-3 fatty acids (nmol/g) in various brain regions of control subjects and subjects with Alzheimer's disease.

n-3 Fatty acid	Control subjects	Subjects with Alzheimer's disease	Adjusted Difference	P-value*
	Mean ± SD ; N	Mean ± SD ; N	(95% CI)	
α-Linolenic (C18:3)				
Temporal cortex	1.75±0.36 ; 17	1.95±0.76 ; 36	0.17 ( −0.22, 0.57 )	0.387
Frontal cortex	2.07±0.42 ; 17	2.46±0.79 ; 37	0.39 ( 0.01, 0.78 )	0.045
Cerebellum	1.65±0.54 ; 16	1.91±0.59 ; 35	0.27 ( −0.08, 0.62 )	0.130
Pooled	1.82±0.47 ; 17	2.12±0.76 ; 37	0.29 ( 0.06, 0.52 )	0.019
Eicosapentaenoic (C20:5)				
Temporal cortex	2.13±1.18 ; 17	1.95±1.34 ; 36	−0.21 ( −0.99, 0.58 )	0.596
Frontal cortex	2.18±1.09 ; 17	2.27±1.57 ; 37	0.08 ( −0.8, 0.97 )	0.848
Cerebellum	1.64±1.11 ; 16	1.46±0.48 ; 35	−0.17 ( −0.62, 0.27 )	0.437
Pooled	1.99±1.13 ; 17	1.9±1.26 ; 37	−0.06 ( −0.59, 0.47 )	0.829
Docosapentaenoic (C22:5)				
Temporal cortex	3.98±2.52 ; 17	3.72±3.23 ; 36	−0.24 ( −2.09, 1.61 )	0.718
Frontal cortex	7.45±1.69 ; 17	7.25±2.41 ; 37	−0.15 ( −1.45, 1.16 )	0.821
Cerebellum	9.45±4.59 ; 16	9.67±2.78 ; 35	0.20 ( −1.87, 2.26 )	0.850
Pooled	7.01±3.81 ; 17	6.84±3.72 ; 37	−0.17 ( −1.28, 0.94 )	0.919

Abbreviations: CI, confidence interval.

*P-values for differences between means were computed by linear regression analysis for each fatty acid in selected brain regions and Generalized Estimating Equations for the pooled analysis in the entire brain after adjustment for age, gender, and post mortem interval.

Figure S1 shows individual data points for docosahexaenoic acid and 1-stearoyl, 2-docosahexaenoyl-*sn*-glycero-3-phosphoethanolamine in temporal cortex from control subjects and Alzheimer's disease patients. The figure also reports the statistical correlation between temporal cortex docosahexaenoic/α-linolenic ratios and most recent MMSE scores (Pearson's correlation coefficient = 0.44, P = 0.005). A comparable correlation was detected between individual MMSE scores and docosahexaenoic/α-linolenic ratios in mid-frontal cortex (Pearson's correlation coefficient = 0.38, P = 0.018), but not in cerebellum (Pearson's correlation coefficient = 0.27, P = 0.12).

Previous reports suggested that low brain levels of docosahexaenoic acid may be accompanied by decreases in n-6 polyunsaturated fatty acids [25], [28], [32]. Here, no significant differences were observed in mid-frontal cortex and cerebellum for free linoleic acid (C18:2n-6), eicosatrienoic acid (C20:3n-6) or arachidonic acid (C20:4n-6) between control subjects and Alzheimer's disease patients (Table S1). A difference was detectable, however, for arachidonic acid in temporal cortex (P = 0.02).

### Docosahexaenoic acid levels in liver

The conversion of α-linolenic acid into docosahexaenoic acid occurs primarily in liver [5], [9], [13]. To examine whether this pathway is altered in Alzheimer's disease, n-3 fatty acids were quantified in liver samples from a second cohort of 9 control subjects and 14 Alzheimer's disease patients. These subjects were selected from a larger available pool because they were negative for hepatitis B or hepatitis C antibodies and, at autopsy, revealed no histological signs of liver disease. A list of medications taken by the subjects is provided in Table S2. There were statistically detectable differences between the two groups for docosahexaenoic acid, eicosapentaenoic acid, docosapentaenoic acid and tetracosahexaenoic acid (Table 3). Additionally, differences were observed for the docosahexaenoic acid-containing phospholipid, 1-O-1′-(*Z*)-octadecenyl, 2-docosahexaenoyl-*sn*-glycero-3-phosphoethanolamine (Table 3).

**Table 3:** Levels of free n-3 fatty acids (nmol/g) and docosahexanoate-containing phosphatidylethanolamine (nmol/g) in liver of control subjects and subjects with Alzheimer's disease.

n-3 Fatty acid	Control subjects	Subjects with Alzheimer's disease	Adjusted Difference	P-value*
	Mean ± SD ; N = 9	Mean ± SD ; N = 14	(95% CI)	
α-Linolenic (C18:3)	28.55±7.88	36.72±22.05	11.19 (−4.53, 26.9)	0.152
Eicosapentaenoic (C20:5)	44.57±14.74	67.4±29.46	27.29 (6.69, 47.88)	0.012
Docosapentaenoic (C22:5)	21.51±8.97	32.39±13.82	12.7 (2.37, 23.03)	0.019
Tetrahexaenoic (C24:6)	0.73±0.17	0.9±0.21	0.19 (0.01, 0.38)	0.041
Docosahexaenoic (C22:6)	324.83±122.89	204.64±74.62	−107.79 (−187.71, −27.87)	0.011
Phosphatidylethanolamine**	75.49±32.30	46.42±15.73	−34.68 (−55.86, −13.50)	0.003

Abbreviations: CI, confidence interval.

*P-values for differences between means were computed by linear regression analysis after adjustment for age, gender, and post mortem interval.

**Phosphatidylethanolamine was 1-O-1′-(*Z*)-octadecenyl, 2-docosahexaenoyl-*sn*-glycero-3-phosphoethanolamine.

**Figure 2:**
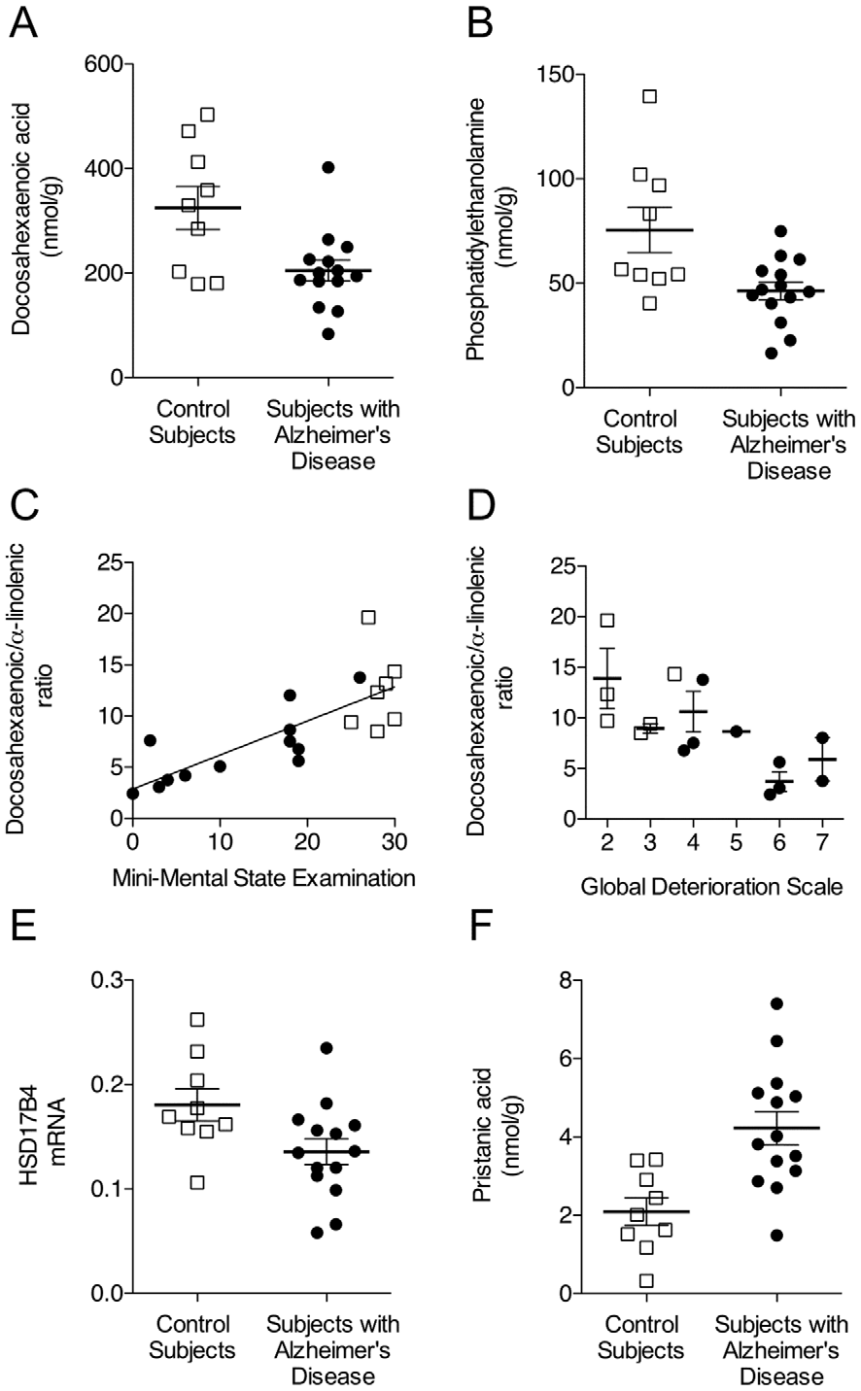
Liver metabolism in Alzheimer's disease patients. Levels of free docosahexaenoic acid (Panel A) and 1-O-1′-(*Z*)-octadecenyl, 2-docosahexaenoyl-*sn*-glycero-3-phosphoethanolamine (Panel B) in liver tissue from control subjects (open squares) and Alzheimer's disease patients (closed circles). Correlation between individual docosahexaenoic/α-linolenic ratios in liver and most recent Mini-Mental State Examination scores (Panel C) or global deterioration scale grades (Panel D). *HSD17B4* mRNA, encoding for d-bifunctional protein (Panel E) and pristanic acid levels (Panel F) in liver from control subjects (open squares) and subjects with Alzheimer's disease (closed circles). Lipid content is expressed in nanomoles per gram of wet tissue and mRNA levels are expressed in arbitrary units. There were statistically detectable differences between control subjects and Alzheimer's disease patients in the levels of docosahexaenoic acid (P = 0.0077) and 1-O-1′-(*Z*)-octadecenyl, 2-docosahexaenoyl-*sn*-glycero-3-phosphoethanolamine (P = 0.003) by two-tailed Welch's *t*-test. There was a significant correlation between docosahexaenoic/α-linolenic ratios in liver and Mini-Mental State Examination scores with use of the partial correlation analysis after adjustment for age, gender and post mortem interval. Global deterioration scale grades correlate significantly (P = 0.013) with the docosahexaenoic/α-linolenic ratios using a linear regression analysis adjusting for age, gender and post mortem interval. There were statistically detectable differences between control subjects and patients in the levels of HSD17B4 mRNA (P = 0.048) and pristanic acid (P = 0.0009) by two-tailed Welch's *t*-test.

Figure 2 shows individual data points for docosahexaenoic acid and 1-O-1′-(*Z*)-octadecenyl,2-docosahexaenoyl-*sn*-glycero-3-phosphoethanolamine in liver, along with correlation analyses between liver docosahexaenoic/α-linolenic ratios and most recent MMSE and global deterioration scale scores. The ratios were positively correlated with MMSE scores (Pearson's correlation coefficient = 0.78, P<0.0001) (Fig. 2C) and negatively correlated with global deterioration scale grades (P = 0.013; Fig. 2D).

No differences were noted for free eicosatrienoic acid and arachidonic acid. In nanomoles per gram of tissue, eicosatrienoic acid was 75.26±33.37 in control subjects and 88.80±39.40 in Alzheimer's disease patients (P = 0.17); and arachidonic acid was 522.46±177.19 in control subjects and 435.96±172.80 in Alzheimer's disease patients (P = 0.46). A significant difference was observed, however, for free linoleic acid, the content of which was, in nanomoles per gram of tissue, 849.00±277.48 in control subjects and 580.17±253.79 in Alzheimer's disease patients (P = 0.042).

### Docosahexaenoic acid biosynthesis in liver

The expression of genes involved in docosahexaenoic acid biosynthesis was measured in the same liver samples. There were statistically detectable differences only for the peroxisomal enzyme d-bifunctional protein (encoded by the *HSD17B4* gene), which catalyzes the conversion of tetracosahexaenoic acid into docosahexaenoic acid [17], [18] (Table 4). Individual data points for *HSD17B4* mRNA are reported in Figure 2E.

**Table 4:** Expression of genes involved in docosahexaenoic acid biosynthesis and peroxisomal function in liver of control subjects and subjects with AD.

Gene	Control Subjects	Subjects with Alzheimer's disease	Adjusted Difference	P-value*
Symbol	Mean ± SD; N = 9	Mean ± SD; N = 14	(95% CI)	
*FADS2*	0.02±0.029	0.016±0.0099	−0.0017 (−0.02, 0.017)	0.851
*HELO1*	0.011±0.012	0.006±0.0046	−0.0043 (−0.012, 0.0036)	0.265
*FADS1*	0.037±0.061	0.024±0.026	−0.0071 (−0.044, 0.03)	0.693
*ABCD1*	0.027±0.034	0.02±0.024	−0.0089 (−0.036, 0.018)	0.500
*ABCD2*	8e-04±0.001	0.0011±0.0013	0.00031 (−8e-04, 0.0014)	0.567
*ACOX1*	0.2±0.24	0.31±0.68	0.069 (−0.45, 0.59)	0.782
*HSD17B4*	0.18±0.045	0.14±0.046	−0.041 (−0.083, −0.00039)	0.048
*PEX13*	0.041±0.017	0.037±0.017	−0.0047 (−0.022, 0.012)	0.569
*PEX14*	0.0087±0.004	0.013±0.022	0.0029 (−0.013, 0.019)	0.700
*PEX19*	0.018±0.018	0.012±0.0054	−0.0055 (−0.016, 0.0051)	0.291

Abbreviations: CI, confidence interval.

*P-values for differences between means were computed by linear regression analysis after adjustment for age, gender, and RNA integrity number.

Hydroxysteroid (17-beta) dehydrogenase 4, *HSD17B4*; for other abbreviations see Text S1.

Since d-bifunctional protein participates in the degradation of branched-chain fatty acids, such as phytanic acid and pristanic acid, the levels of these compounds were also measured. In nanomoles per gram of tissue, phytanic acid was 1.93±0.51 in control subjects and 4.33±1.80 in Alzheimer's disease patients [adjusted difference (95% confidence intervals): 2.53 (1.17, 3.90); P = 0.001]; pristanic acid was 2.10±1.05 in control subjects and 4.23±1.58 in Alzheimer's disease patients [adjusted difference (95% confidence intervals): 2.37 (1.03, 3.70); P = 0.002]. Individual data points for pristanic acid are shown in Figure 2F.

## Discussion

The first objective of this study was to reexamine the association, suggested by past reports [6], [24]–[30], between Alzheimer's disease and lowered brain docosahexaenoic acid content. Our findings provide new evidence in support of such association and further reveal the existence of a positive correlation between brain docosahexaenoic acid levels and cognitive status. To identify potential mechanisms responsible for the observed breakdown in brain docosahexaenoic acid integrity, we next focused our attention on the liver because of the critical role played by this organ in supplying docosahexaenoic acid to the brain [5], [9], [13]. Our results indicate that docosahexaenoic acid levels and expression of d-bifunctional protein, a key enzyme of docosahexaenoic acid biosynthesis, are selectively reduced in liver of Alzheimer's disease patients. The functional significance of these findings is underscored by the identification of a strong positive correlation between liver docosahexaenoic acid content and cognitive status (P<0.0001).

Previous studies have suggested that brain levels of docosahexaenoic acid [6] and docosahexaenoic acid-containing phospholipids [24]–[30] are reduced in Alzheimer's disease (see [31] for a discussion of discordances). Our investigations confirmed those results. In agreement with Lukiw et al. (2005) [6], we found that docosahexaenoic acid is reduced in temporal cortex and mid-frontal cortex from 37 subjects with Alzheimer's disease, compared to 17 closely matched control subjects. Additionally, our analyses suggested that (*i*) this deficit is selective for docosahexaenoic acid, because other n-3 or n-6 fatty acids are only marginally affected, and (*ii*) docosahexaenoic acid levels in temporal cortex and mid-frontal cortex positively correlate with cognitive status. This correlation highlights the significance of brain docosahexaenoic acid in normal cognition, though further research is needed to determine if it represents cause and effect.

We were surprised to find that a decrease in docosahexaenoic acid was clearly detectable in cerebellum, a brain region that is regarded as being less vulnerable to Alzheimer's pathology. This finding led us to hypothesize that the alteration in brain docosahexaenoic acid might result from a systemic deficiency in the biosynthesis of this fatty acid. In mammals, the enzyme pathway responsible for docosahexaenoic acid production is primarily localized to the liver [5], [9], [13]. Therefore, to determine whether this pathway is dysfunctional in Alzheimer's disease, we examined the lipid composition of liver samples from 14 patients and 9 control subjects, who displayed no histological sign of liver pathology. Our analyses show that liver tissue from Alzheimer's patients contains reduced levels of docosahexaenoic acid, but elevated levels of tetracosahexaenoic acid and other n-3 fatty acids. This profile is incompatible with a nutritional deficit in n-3 fatty acids and is suggestive of a defect in the last step of docosahexaenoic acid biosynthesis – the β-oxidative conversion of tetracosahexaenoic acid into docosahexaenoic acid, which occurs in liver peroxisomes (Fig. 1). Two additional findings support this interpretation and point to a selective involvement of peroxisomal d-bifunctional protein: the accumulation of pristanic acid and phytanic acid, two substrates for liver d-bifunctional protein, and the lowered expression of the *HSD17B4* gene, which encodes for this protein [17], [18], [33]. Notably, other genes included in our panel were not significantly different between Alzheimer's disease patients and control subjects. These results are consistent with previous reports suggesting that d-bifunctional protein mutations are associated with reduced docosahexaenoic acid levels in human liver and brain [34], [35].

The pathological events that lead to down-regulation of liver d-bifunctional protein in Alzheimer's disease remain to be discovered. However, a role for oxidative stress, which is known to accelerate age-dependent liver peroxisomal damage [36]–[38], might be hypothesized. Furthermore, it is important to note that our analyses were focused on docosahexaenoic acid biosynthesis and did not evaluate the potential impact of other aspects of docosahexaenoic acid metabolism – including transport and oxidation.

A significant outcome of our analyses was the discovery that liver docosahexaenoic acid levels are positive correlated with MMSE scores, and negatively correlated with global deterioration scale grades. Although it is well established that patients with advanced liver diseases show a decline in cognitive abilities [39], these findings reveal a previously unrecognized association between hepatic docosahexaenoic acid homeostasis and global cognition, and suggest that subtle changes in liver docosahexaenoic acid metabolism might be associated with dementia. This supports early suggestions that in neurodegenerative disorders and aging, a failure in the supply of docosahexaenoic acid from the liver may take place [9]. Moreover, a circulatory “long loop” connects the supply of docosahexaenoic acid to the biogenesis of excitable and photoreceptor membranes [40]. In retinal degenerative diseases, a shortage of blood docosahexaenoic acid has been demonstrated, and a failure of the “long loop” from the liver is suggested to underlie these changes [41]. Future prospective investigations should explore the temporal relationship between such changes and the development of Alzheimer's pathology. As with all observational studies, there is the possibility that unmeasured factors may explain our results, even though we chose a priori to adjust for the three most likely confounders – age, gender and post mortem interval. Furthermore, though limited by the lack of information on dosages and treatment duration, our analysis of the medication record reveals no obvious correlation between individual drug classes and liver docosahexaenoic acid (Figure S2). Despite these inevitable limitations, our results do suggest that a dysfunction in liver docosahexaenoic acid metabolism, at least partly caused by abnormal d-bifunctional protein expression, might predispose persons to Alzheimer's disease. This has implications both for clinical interventions with n-3 fatty acids, which should take into consideration the limited ability of Alzheimer's disease patients to complete docosahexaenoic acid biosynthesis, and for the discovery of peripheral lipid biomarkers of Alzheimer's disease.

## Materials and Methods

### Study design and tissue procurement

We conducted this study in two parts. We first determined whether brain levels of docosahexaenoic acid are altered in Alzheimer's disease [6], [24]–[30] and whether such alterations correlate with cognitive status. We used frozen brain samples from a total of 17 non-demented control subjects and 37 pathologically confirmed subjects with Alzheimer's disease (males/females: control subjects, 10/7; subjects with Alzheimer's disease, 20/17), provided by the Institute for Brain Aging and the Dementia and Alzheimer's Disease Research Center at the University of California, Irvine. Three brain areas were selected for analysis: temporal cortex (Broadmann area 20; 17 control subjects and 36 subjects with Alzheimer's disease), mid-frontal cortex (Broadmann area 9; 17 control subjects and 37 subjects with Alzheimer's disease), and cerebellum (16 control subjects and 35 subjects with Alzheimer's disease). Subjects were matched for age (in years: control subjects, 80.5±8.5; subjects with Alzheimer's disease, 80.5±7.3) and post mortem interval (in hours: control subjects, 4.4±1.5; subjects with Alzheimer's disease, 4.2±1.7). Alzheimer's disease cases met the National Institute on Aging-Reagan Institute criteria for intermediate or high likehood of Alzheimer's disease. Mini Mental State Examination (MMSE) scores, a measure of cognitive status, were available for 10 control subjects (mean score±SD = 28.3±1.8; assessed 44.3±35.9 months before death) and 29 subjects with Alzheimer's disease (mean score±SD = 12.4±7.2; assessed 11.0±6.2 months before death).

Because the liver is a primary source of brain docosahexaenoic acid, in the second part of the study we examined whether Alzheimer's disease might be associated with alterations in liver n-3 fatty acid metabolism. Frozen liver samples from a separate cohort of 9 control subjects and 14 subjects with Alzheimer's disease (males/females: control subjects, 7/2; subjects with Alzheimer's disease, 8/6) were obtained from the Banner Sun Health Research Institute (Sun City, AZ). These were selected from a larger available pool because they were negative for hepatitis B or hepatitis C antibodies and, at autopsy, revealed no histological signs of liver disease (by hematoxylin/eosin staining). They were matched for age (in years: control subjects, 83.9±5.4; subjects with Alzheimer's disease, 84.6±6.7) and post mortem interval (in hours: control subjects, 3.4±0.9; subjects with Alzheimer's disease, 3.2±0.6). Alzheimer's disease cases met the National Institute on Aging-Reagan Institute criteria for intermediate or high likelihood of Alzheimer's disease. MMSE scores were available for 7 control subjects (mean±SD = 28.1±1.7; assessed 7.1±5.6 months before death) and 12 subjects with Alzheimer's disease (mean±SD = 11.9±10.6; assessed 10.1±8.7 months before death). Global deterioration scale scores were available for 6 control subjects and 9 subjects with Alzheimer's disease.

All subjects and their caregivers (when appropriate) provided written informed consent for both the clinical examination as well as for brain donation at the University of California Irvine and for liver donation at the Banner Sun Health Research Institute Brain and Body Donation Program. The protocols and informed consent have been approved by the University of California Irvine Institutional Biosafety Committee and the Banner Health Institutional Review Board.

### Lipid and gene expression analyses

Analyses are described in Text S1 available online with this article.

### Statistical analyses

Descriptive statistics are presented as means ± SD. The differences between unadjusted mean values were determined by two-tailed Welch's *t*-test. Associations between parameters were tested by partial correlation analysis (Pearson's). Generalized Estimating Equations [42] were used to determine the overall association of individual lipid species with Alzheimer's disease – adjusting for the a priori-specified potential confounders of age, gender, and post mortem interval – by pooling data across frontal cortex, temporal cortex and cerebellum. An exchangeable working correlation structure was used in model fitting, and robust standard errors [43] were used for inference in the Generalized Estimating Equations analysis. Adjusting for the covariates listed above, we used linear regression to estimate the association between individual lipid species and Alzheimer's disease. Linear regression was also utilized to determine differences in mRNA expression levels adjusting for age, gender, and RNA integrity number. All confidence intervals correspond to a 95% confidence level without adjustment for multiple comparisons.

## Supporting Information

Text S1 Supplementary Materials and Methods.
(0.06 MB DOC)

Figure S1 Levels of free DHA (Panel A) and 1-stearoyl-2-docosahexaenoyl-sn-glycero-3-phosphoethanolamine (Panel B) in temporal cortex of control subjects (open squares) and subjects with AD (closed circles). Correlation analysis between individual docosahexaenoic/α-linolenic ratios in temporal cortex and most recent Mini-Mental State Examination scores (Panel C). Lipid content is expressed in nanomoles per gram of wet tissue. There were statistically detectable differences between control subjects and patients in the levels of DHA (P = 0.019) and 1-stearoyl-2-docosahexaenoyl-sn-glycero-3-phosphoethanolamine (P = 0.0084) by two-tailed Welch's t-test. There was a significant correlation between docosahexaenoic/α-linolenic ratios and Mini-Mental State Examination scores by partial correlation analysis after adjustment for age, gender and post mortem interval.
(6.78 MB TIF)

Figure S2 Correlation between liver docosahexaenoic acid and individual drug classes taken by the control subjects (open squares) and Alzheimer's disease patients (closed circles).
(6.78 MB TIF)

Table S1 Levels of free n-6 fatty acids (nmol/g) in various brain regions of control subjects and subjects with Alzheimer's disease.
(0.10 MB DOCX)

Table S2 List of medications taken by the subjects involved in the liver study.
(0.06 MB DOCX)
