# Supplementary material for: Deficient Liver Biosynthesis of Docosahexaenoic Acid Correlates with Cognitive Impairment in Alzheimer's Disease
Source: PLoS One. 2010 Sep 8;5(9):e12538. doi: 10.1371/journal.pone.0012538 (PMC2935886; doi:10.1371/journal.pone.0012538)
Supplement: Text S1 — Supplementary Materials and Methods. (0.06 MB DOC) [file pone.0012538.s001.doc]

**Supplementary text**

**Lipid analyses.**Lipid analyses were conducted as described [1]. Briefly, frozen tissue samples were weighed and homogenized in cold methanol containing appropriate authentic standards (listed below). Lipids were extracted by adding chloroform and water (2/1, vol/vol) and fractionated through open-bed silica gel columns by progressive elution with chloroform/methanol mixtures. Fractions eluted from the columns were dried under nitrogen, reconstituted in chloroform/methanol (1:4, vol/vol; 0.1 ml) and subjected to liquid chromatography/mass spectrometry. Fatty acids were quantified with an Agilent 1100 liquid chromatograph coupled to a 1946D mass detector equipped with an electrospray ionization interface (Agilent Technologies, Palo Alto, CA). A reversed-phase XDB Eclipse C18 column (50x4.6 mm i.d., 1.8 μm, Zorbax, Agilent Technologies) was eluted with a linear gradient from 90% to 100% of A in B for 2.5 min at a flow rate of 1.5 ml/min with column temperature at 40˚C. Mobile phase A consisted of methanol containing 0.25% acetic acid and 5 mM ammonium acetate; mobile phase B consisted of water containing 0.25% acetic acid and 5 mM ammonium acetate. Column temperature was kept at 40˚C. Mass detection was in the negative ionization mode, capillary voltage was set at 4.0 kV and fragmentor voltage was 120 V. Nitrogen was used as drying gas at a flow rate of 13 liters/min and a temperature of 350˚C. Nebulizer pressure was set at 60 pounds per square inch. For quantification purposes, the deprotonated pseudo-molecular ions [M-H]^-^ of the fatty acids were monitored in the selected ion-monitoring mode, using [^2^H_8_]-arachidonic acid (Cayman Chemical, Ann Arbor, MI) as internal standard (mass-to-charge ratio, *m/z*= 311.3). The following tissue lipids were quantified: α-linolenic acid (*all-cis*-9,12,15-octadecatrienoic acid, *m/z*= 277.3), eicosapentaenoic acid (*all-cis*-5,8,11,14,17-eicosapentaenoic acid, *m/z*= 301.3), docosapentaenoic acid (*all-cis-*7,10,13,16,19-docosapentaenoic acid, *m/z*= 329.3), docosahexaenoic acid (*all-cis*-4,7,10,13,16,19-docosahexaenoic acid, *m/z*= 327.3), tetracosahexaenoic acid (*all-cis*-6,9,12,15,18,21-tetracosahexaenoic acid, *m/z*= 355.3), linoleic acid (*all-cis*-9,12-octadecadienoic acid, *m/z*= 279.3), dihomo-gamma-linolenic acid (*all-cis*-8,11,14-eicosatrienoic acid, *m/z*= 305.3), arachidonic acid (*all-cis*-5,8,11,14-eicosatetraenoic acid, *m/z*= 303.3), phytanic acid (3,7,11,15-tetramethyl hexadecanoic acid, *m/z*= 311.3), and pristanic acid (2,6,10,14-tetramethylpentadecanoic acid, *m/z*= 297.3). Commercially available fatty acids (Nu-Chek Prep, Elysian, MN; Cayman Chemical or Sigma-Aldrich, St Louis, MO) were used as references. Phosphatidylethanolamine species containing docosahexaenoic acid were identified by tandem mass spectrometry, using an Agilent 1100 liquid chromatograph coupled to an electrospray ionization-ion-trap XCT mass detector. A reversed-phase Poroshell 300SB C18 column (2.1x75 mm i.d., 5 μm, Agilent) was eluted with a linear gradient from 85% to 100% of mobile phase A in B in 5 min at a flow rate of 1.0 ml/min with column temperature at 50˚C. Mobile phase composition was as described above. The capillary voltage was set at 4.0 kV and skimmer voltage at -40 V. Nitrogen was utilized as drying gas at a flow rate of 10 liters/min, temperature at 350˚C and nebulizer pressure at 60 pounds per square inch. Helium was the collision gas and fragmentation amplitude was set at 1.2 V. Mass detection was in the negative ionization mode and was controlled by the Agilent/Bruker Daltonics software version 5.2.  In brain samples, 1-stearoyl, 2-docosahexaenoyl-*sn*-glycero-3-phosphoethanolamine was quantified by multiple reaction monitoring of the transition *m/z*=790.5>480.3. In liver samples, 1-O-1'-(*Z*)-octadecenyl, 2-docosahexaenoyl-*sn*-glycero-3-phosphoethanolamine was quantified monitoring the transition *m/z*=774.5>464.3. In both cases, synthetic 1,2-diheptadecanoyl-*sn*-glycero-3-phosphoethanolamine (Avanti Polar Lipids, Alabaster, AL) was used as internal standard, monitoring the transition *m/z*=718.5>466.3.

**Gene expression.** Total RNA was extracted from 10–200 mg of frozen liver tissue using TRIzol reagent (Invitrogen, Carlsbad, CA) and was purified with the RNeasy mini kit (Qiagen, Valencia, CA).  RNA quality was assessed using an Agilent BioAnalyzer and by UV spectrophotometry. First-strand complementary DNAs were synthesized using SuperScript II RNase H reverse transcriptase (Invitrogen). Reverse transcription of total RNA (2 µg) was carried out using oligo(dT)12–18 primers for 50 min at 42°C. mRNA levels were measured by quantitative real-time polymerase chain reaction with a Mx 3000P system (Stratagene, La Jolla, CA). The following primers and fluorogenic probes were purchased from Applied Biosystems (TaqMan Gene Expression Assays, Foster City, CA): acyl-coenzyme A oxidase 1 (*ACOX1*, Hs01074245_m1), hydroxysteroid (17-beta) dehydrogenase 4 (*HSD17B4*, Hs01069908_m1), peroxisome biogenesis factor-13 (*PEX13*, Hs00159996_m1), peroxisome biogenesis factor-14 (*PEX14*, Hs00190073_m1), ATP-binding cassette, sub-family D member 1 (*ABCD1*, Hs01110018_m1), ATP-binding cassette, sub-family D, member 2 (*ABCD2*, Hs01115621_m1), ribosomal protein S9 (*RPS9*, Hs02339426_g1). Primers and fluorogenic probes for Δ^5^ desaturase (*FADS1,* fatty acid desaturase-1), Δ^6^ desaturase (*FADS2,* fatty acid desaturase-2), elongase (*HELO1*), and peroxisome biogenesis factor-19 (*PEX19*) were synthesized at TIB MOLBIOL GmbH (Adelphia, NJ) utilizing primers and probes sequences previously published [2]. Each probe was conjugated to an FAM reporter at the 5’ end and a TAMRA quencher at the 3’ end.  RNA levels were normalized using *RPS9* as standard [3].

Supplementary References

1. Astarita G, Ahmed F, Piomelli D (2009) Lipidomic analysis of biological samples by liquid chromatography coupled to mass spectrometry. Methods Mol Biol 579: 201-219.

2. McNamara RK, Liu Y, Jandacek R, Rider T, Tso P (2008) The aging human orbitofrontal cortex: decreasing polyunsaturated fatty acid composition and associated increases in lipogenic gene expression and stearoyl-CoA desaturase activity. Prostaglandins Leukot Essent Fatty Acids 78: 293-304.

3. Janovick-Guretzky NA, Dann HM, Carlson DB, Murphy MR, Loor JJ, et al. (2007) Housekeeping gene expression in bovine liver is affected by physiological state, feed intake, and dietary treatment. J Dairy Sci 90: 2246-2252.
