# Supplementary figures and images for: Deficient Liver Biosynthesis of Docosahexaenoic Acid Correlates with Cognitive Impairment in Alzheimer's Disease
Source: PLoS One. 2010 Sep 8;5(9):e12538. doi: 10.1371/journal.pone.0012538 (PMC2935886; doi:10.1371/journal.pone.0012538)

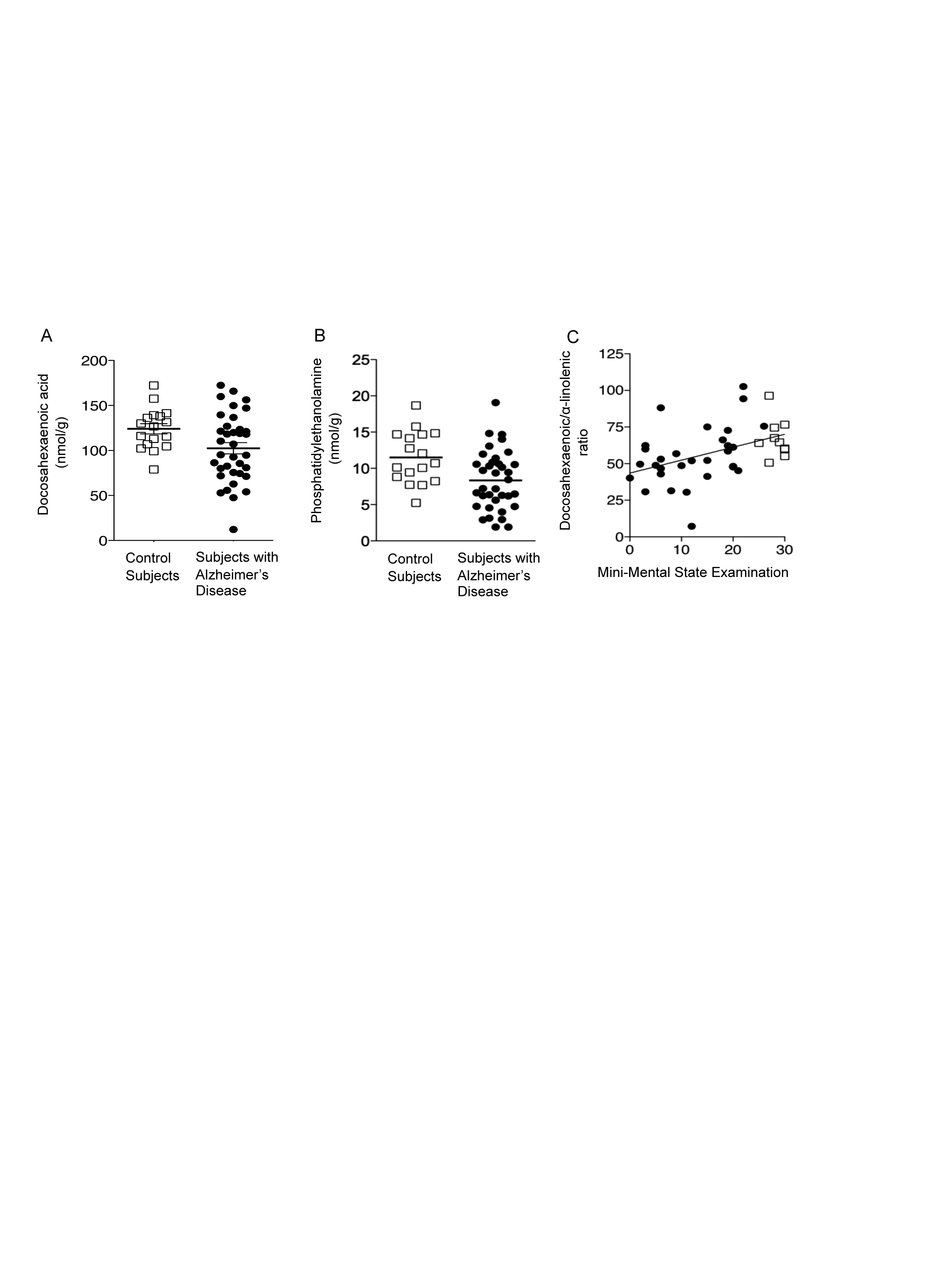

Supplement: Figure S1 — Levels of free DHA (Panel A) and 1-stearoyl-2-docosahexaenoyl-sn-glycero-3-phosphoethanolamine (Panel B) in temporal cortex of control subjects (open squares) and subjects with AD (closed circles). Correlation analysis between individual docosahexaenoic/α-linolenic ratios in temporal cortex and most recent Mini-Mental State Examination scores (Panel C). Lipid content is expressed in nanomoles per gram of wet tissue. There were statistically detectable differences between control subjects and patients in the levels of DHA (P = 0.019) and 1-stearoyl-2-docosahexaenoyl-sn-glycero-3-phosphoethanolamine (P = 0.0084) by two-tailed Welch's t-test. There was a significant correlation between docosahexaenoic/α-linolenic ratios and Mini-Mental State Examination scores by partial correlation analysis after adjustment for age, gender and post mortem interval. (6.78 MB TIF) [file pone.0012538.s002.tif]

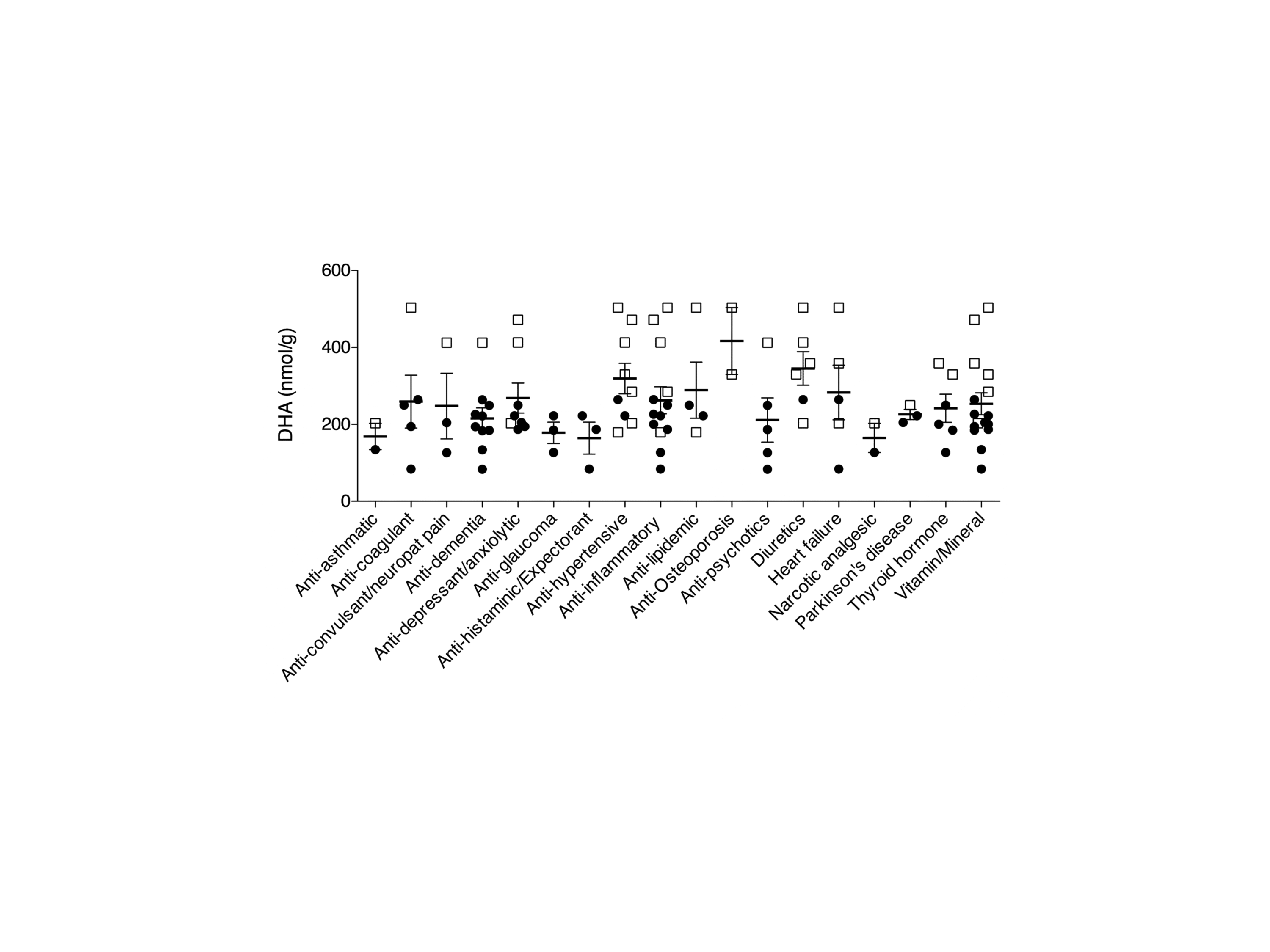

Supplement: Figure S2 — Correlation between liver docosahexaenoic acid and individual drug classes taken by the control subjects (open squares) and Alzheimer's disease patients (closed circles). (6.78 MB TIF) [file pone.0012538.s003.tif]
