# Supplementary material for: Deficient Liver Biosynthesis of Docosahexaenoic Acid Correlates with Cognitive Impairment in Alzheimer's Disease
Source: PLoS One. 2010 Sep 8;5(9):e12538. doi: 10.1371/journal.pone.0012538 (PMC2935886; doi:10.1371/journal.pone.0012538)
Supplement: Table S1 — Levels of free n-6 fatty acids (nmol/g) in various brain regions of control subjects and subjects with Alzheimer's disease. (0.10 MB DOCX) [file pone.0012538.s004.docx]

| n-6 Fatty acid | Control subjects | Subjects with Alzheimer’s disease | Adjusted Difference | P-value^*^ |
| --- | --- | --- | --- | --- |
|  | Mean ± SD ; N | Mean ± SD ; N | (95% CI) |  |
| Linoleic (C18:2) |  |  |  |  |
| Temporal cortex | 28.07 ± 6.6 ; 17 | 25.44 ± 7.58 ; 36 | -2.81 (-7.22, 1.59) | 0.205 |
| Frontal cortex | 27.98 ± 6.97 ; 17 | 29.12 ± 7.13 ; 37 | 1.26 (-2.71, 5.24) | 0.527 |
| Cerebellum | 26.03 ± 6.82 ; 16 | 25.71 ± 6.8 ; 35 | -0.31 (-4.5, 3.89) | 0.883 |
| Pooled | 27.39 ± 6.72 ; 17 | 26.79 ± 7.31 ; 37 | -0.63 (-3.36, 2.1) | 0.65 |
| Eicosatrienoic (C20:3) |  |  |  |  |
| Temporal cortex | 25.18 ± 10.96 ; 17 | 27.31 ± 9.99 ; 36 | 1.49 (-4.56, 7.54) | 0.623 |
| Frontal cortex | 20.86 ± 3.65 ; 17 | 29.87 ± 12.32 ; 37 | 8.91 (3.06, 14.75) | 0.004 |
| Cerebellum | 27.53 ± 12.33 ; 16 | 25.21 ± 7.04 ; 35 | -2.3 (-7.76, 3.17) | 0.402 |
| Pooled | 24.46 ± 9.89 ; 17 | 27.51 ± 10.15 ; 37 | 2.86 (-1.26, 6.98) | 0.174 |
| Arachidonic (C20:4 ) |  |  |  |  |
| Temporal cortex | 289.11 ± 75.22 ; 17 | 248.16 ± 67.47 ; 36 | -47.42 (-86.97, -7.86) | 0.02 |
| Frontal cortex | 259.8 ± 71.86 ; 17 | 249.51 ± 63.86 ; 37 | -10.42 (-46.61, 25.78) | 0.566 |
| Cerebellum | 338.52 ± 121.41 ; 16 | 302.42 ± 72 ; 35 | -35.98 (-90.5, 18.55) | 0.191 |
| Pooled | 294.96 ± 95.43 ; 17 | 266.21 ± 71.72 ; 37 | -30.49 (-59.35, -1.64) | 0.038 |

**Table S1.** Levels of free n-6 fatty acids (nmol/g) in various brain regions of control subjects and subjects with Alzheimer’s disease.

Abbreviations: CI, confidence interval.

^*^ P-values for differences between means were computed by linear regression analysis for each fatty acid in selected brain regions and Generalized Estimating Equations for the pooled analysis in the entire brain after adjustment for age, gender, and post mortem interval.
