# Supplementary material for: Deficient Liver Biosynthesis of Docosahexaenoic Acid Correlates with Cognitive Impairment in Alzheimer's Disease
Source: PLoS One. 2010 Sep 8;5(9):e12538. doi: 10.1371/journal.pone.0012538 (PMC2935886; doi:10.1371/journal.pone.0012538)
Supplement: Table S2 — List of medications taken by the subjects involved in the liver study. (0.06 MB DOCX) [file pone.0012538.s005.docx]

**Table S2**. List of medications taken by the subjects involved in the liver study.

| Category | Drugs |
| --- | --- |
| Anti-asthmatic | Albuterol  Ipratropium bromide |
| Anti-convulsant/neuropatic pain | Gabapentin  Phenytoin  Valproate semisodium |
| Anti-coagulant | Aspirin (heart)  Warfarin |
| Anti-dementia | Donepezil  Galantamine  Memantine  Rivastigmine  Selegiline |
| Anti-depressant/anxiolytic | Alprazolam  Diazepam  Escitalopram  Fluoxetine  Lorazepam  Paroxetine  Sertraline hydrochloride  Temazepam |
| Anti-glaucoma | Brimonidine  Dorzolamide  Latanoprost |
| Anti-histaminic/Expectorant | Diphenhydramine hydrochloride  Famotidine  Fexofenadine  Hydroxyzine  Loratadine |
| Anti-hypertensive | Amlodipine  Atenolol  Diltiazem  Isosorbide Mononitrate  Lisinopril  Losartan  Metoprolol  Terazosin  Verapamil |
| Anti-inflammatory | Acetaminophen  Celecoxib  Etodolac  Fluticasone  Ibuprofen  Ketoprofen  Prednisolone  Valdecoxib |
| Anti-lipidemic | Atorvastatin  Rosuvastatin |
| Anti-psychotic | Olanzapine  Ziprasidone  Valproate semisodium |
| Anti-Osteoporosis | Alendronate  Risedronate |
| Diuretics | Furosemide  Hydrochlorothiazide  Triamterene |
| Heart failure | Carvedilol  Digoxin |
| Narcotic analgesic | Hydrocodone  Oxycodone |
| Parkinson's disease | Carbidopa/Levodopa  Entacapone  Pramipexole |
| Thyroid | Levothyroxine |
| Vitamin/Mineral | Calcium  Coenzyme Q10  Folic Acid  Multi-Vitamins  Potassium Chloride  Selenium  Vitamin B Complex  Vitamin B6  Vitamin C  Vitamin D  Vitamin E  Zinc |
